# The Ubiquitin-Proteasome System in Apoptosis and Apoptotic Cell Clearance

**DOI:** 10.3389/fcell.2022.914288

**Published:** 2022-07-06

**Authors:** Lei Yuan, Peiyao Li, Qian Zheng, Hui Wang, Hui Xiao

**Affiliations:** College of Life Sciences, Shaanxi Normal University, Xi’an, China

**Keywords:** ubiquitin, E3 ligase, apoptosis, apoptotic cell clearance, deubiquination

## Abstract

Ubiquitination, a critical post-translational modification of proteins, refers to the covalent attachment of ubiquitin to the substrate and is involved in various biological processes such as protein stability regulation, DNA damage repair, and apoptosis, among others. E3 ubiquitin ligases are essential enzymes of the ubiquitin pathway with high substrate specificity and precisely regulate specific proteins’ turnover. As one of the most well-studied forms of programmed cell death, apoptosis is substantially conserved across the evolutionary tree. The final critical stage in apoptosis is the removal of apoptotic cells by professional and non-professional phagocytes. Apoptosis and apoptotic cell clearance are crucial for the normal development, differentiation, and growth of multicellular organisms, as well as their association with a variety of inflammatory and immune diseases. In this review, we discuss the role of ubiquitination and deubiquitination in apoptosis and apoptotic cell clearance*.*

## Introduction

Protein renewal is rigorously regulated in organisms where proteins participate in various biological activities, and about one-third of freshly generated proteins are swiftly degraded (Schubert et al., 2000). Protein post-translational modifications are required for cells to maintain homeostasis and adapt rapidly to environmental changes. Ubiquitination is a reversible post-translational modification of proteins that is catalyzed by the E1 activating enzyme, the E2 conjugating enzyme, and the E3 ligase. It involves the covalent binding of a small protein called ubiquitin, which contains 76 amino acids, to the lysine residues of a target protein (Smit and Sixma, 2014). Ubiquitination is a dynamically reversible process in which ubiquitin is eliminated by deubiquitinases (DUB) (Clague et al., 2013). Ubiquitination participates in a variety of physiological activities, including cell cycle progression, protein quality control, transcriptional regulation (Mukhopadhyay and Riezman, 2007), DNA damage repair (Ulrich and Walden, 2010), apoptosis, and vesicle trafficking (Vucic et al., 2011).

Apoptosis, one of the most well-studied forms of programmed cell death, is substantially conserved across the evolutionary tree from lower creatures (*Caenorhabditis elegans* and *Drosophila melanogaster*) to mammals. The final critical stage in apoptosis is the removal of apoptotic cells by professional and non-professional phagocytes. When cells undergo apoptosis, phosphatidylserine (PS) is exposed to the cell surface, where it is recognized as a “eat-me” signal by phagocytic receptors on phagocytes, activating downstream phagocytic pathways, which result in the internalization of apoptotic cells and formation of phagosomes (Nagata et al., 2010; Zheng et al., 2021). Phagosomes then undergo early and late stages before fusing with lysosomes to form phagolysosomes for apoptotic cell degradation. The timely and effective removal of apoptotic cells is vital for organisms to maintain the homeostasis of the intracellular environment and avoid inflammatory responses (Nagata, 2018).

The role of ubiquitination in apoptosis and the clearance of apoptotic cells will be discussed in this review. We describe how ubiquitination, the most complicated and critical post-translational modification of proteins, alters and impacts the localization, degradation, signaling, and other functions of crucial components in apoptosis and the clearance of apoptotic cells, further demonstrating the importance of studying ubiquitination in apoptosis and the clearance of apoptotic cells.

### General Overview of Ubiquitination Modifications

**FIGURE 1:**
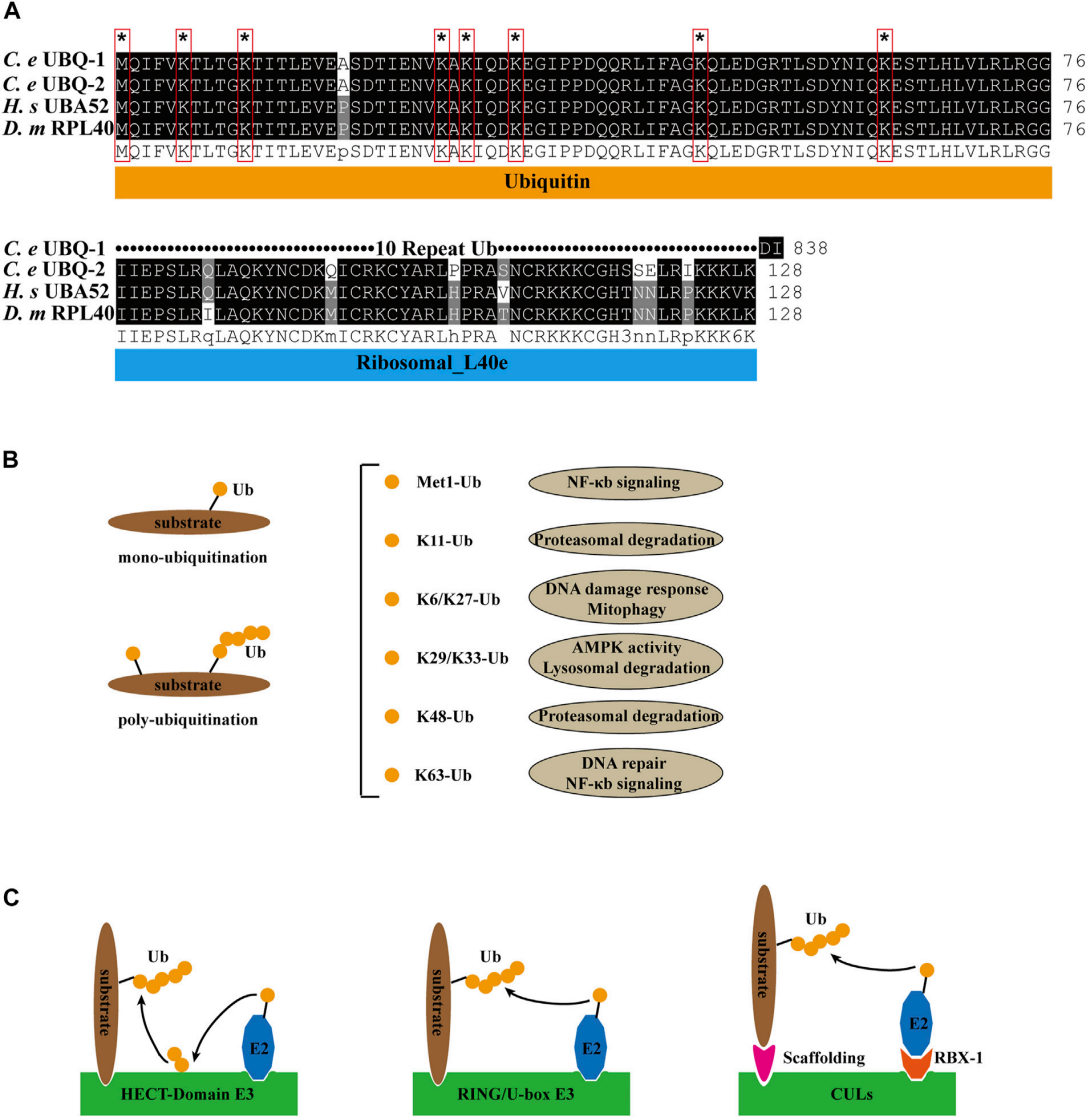
Ubiquitin pathway compounds and functions. **(A)** Sequence alignment of *C. elegans* (*C. e*) UBQ-1, UBQ-2, human (*H. s*) UBA52 and *Drosophila melanogaster* (*D. m*) RPL40. Identical residues are shaded in black, and similar ones in gray. * and red boxes indicate ubiquitin lysine residue site. **(B)** Ubiquitin linkage chains and functions. **(C)** Structural model of HECT-domain, RING/U-box and CULs E3 complex.

As a small molecule protein, ubiquitin exists widely in all eukaryotic cells and has a highly conserved sequence, with only three amino acid differences between lower organisms and mammals. There are two ubiquitin genes in *C. elegans*: *ubq-1* and *ubq-2*. UBQ-1 contains 11 tandem Ub (Graham et al., 1989). *ubq-2* is orthologous to human UBA52 and *Drosophila melanogaster* RPL40, and it encodes the Ub and L40 ribosomal large subunit proteins (Finley et al., 1989) (Figure 1A). All seven lysine residues (K6, K11, K27, K29, K33, K48, and K63) and the methionine (Met1) site at the N terminus of ubiquitin can be ubiquitinated to expand the ubiquitin chain. Various linkages between ubiquitin chains and substrates lead to different fates of substrates, like K48-Ub mediates protein degradation, K63-Ub mediates DNA repair, NF-κb signaling (Saeki et al., 2009; Ohtake et al., 2016), K11-Ub mediates proteasomal degradation, K6/K27-Ub relates to DNA damage response and mitophagy (Michel et al., 2017), K29/K33-Ub inhibits AMPK activity (Michel et al., 2015; Kaiho-Soma et al., 2021), and Met1-Ub regulates NF-κb signaling (Hrdinka et al., 2016) (Figure 1B).

E1 (*uba-1* in *C. elegans*, an ortholog of human *uba1*) consumes ATP to form acyl-adenylate complexes, which activate the C-terminus of ubiquitin (Maeda et al., 2001). Activated ubiquitin forms a thioester bond with the cysteine residue of E2 (E2s in both *C. elegans*, *D. melanogaster*, and *H. sapiens* are listed in Table 1) (Jones et al., 2002). Finally, the activated ubiquitin is transferred to the substrate directly or by E3. mono- and poly-ubiquitination are categorized based on the substrate-binding ubiquitin molecules (Figure 1B). According to the structural characteristics of E3, four distinct types can be defined. HECT domain E3s transfer ubiquitin from E2 to the cysteine residues of E3sand then transfer Ub to the substrates. E3s with RING and U-box structural domains bind to both ubiquitin-containing E2 and the substrate, transferring ubiquitin to the substrate. CULs form a rigid scaffold with the C-terminus binding RING-H2 finger protein Rbx1/Roc1 and the N-terminal binding junction Skp1, which recognizes with the substrate through the F-box motif of SRS, whereas other CULs have a similar scaffolding function (Zheng et al., 2002; Fang et al., 2003) (Figure 1C). As a dynamically reversible process, deubiquitinating enzymes (DUBs) maintain an equilibrium of ubiquitination. DUBs have been classified into two categories: cysteine proteases and metalloproteases (Nijman et al., 2005).

**TABLE 1:** Ubiquitin-conjugating enzymes (E2) in *C. elegans*, *D. melanogaster* and *H. sapiens*.

Species No.	*Caenorhabditis elegans*	*Drosophila melanogaster*	*Homo sapiens*
1	*ubc-1*	*UbcD6*	*UBE2B*
2	*ubc-2*	*Dsi/Ubc1*	*UBE2D2/UBE2D3*
3	*ubc-3*	*CG7656*	*CDC34/UBE2R2*
4	*ubc-6*	*CG5823*	*NCUBE1*
5	*ubc-7*	*CG9602*	*UBE2G1*
6	*ubc-8*	*CG2257/CG14739*	*UBE2H*
7	*ubc-9*	*CG3018*	*UBE2I*
8	*ubc-12*	*CG7375*	*UBE2M*
9	*ubc-13*	*CG3473*	*UBE2N*
10	*ubc-14*	*CG4443*	*UBE2G2*
11	*ubc-15*	*CG5823*	*NCUBE1*
12	*ubc-16*	*CG7220*	*UBE2W*
13	*ubc-17*	*CG6303*	*BIRC6*
14	*ubc-18*	*CG17030*	*UBE2L3*
15	*ubc-19*	*CG46338*	*AKTIP*
16	*ubc-20*	*UbcD4*	*UBE2K*
17	*ubc-21*	*UbcD4*	*UBE2K*
18	*ubc-22*	*CG17030*	*UBE2L1*
		*UbcD10*	*UBE2L3/UBCH7*
		*Ubc84D*	*UBE2L6*
19	*ubc-23*	*UbcD4*	*FAF1*
20	*ubc-24*	*—*	*—*
21	*ubc-25*	*CG2924*	*UBE2Q1/UBE2Q2*
22	*ubc-26*	*CG5823*	*NCUBE1*

### The Apoptosis and Apoptotic Cell Clearance

The process of apoptosis can be classified into several key steps, including death signal initiation and transmission, death program triggering, and the clearance of apoptotic cells (Wang and Yang, 2016; Zheng et al., 2017). In the preceding ten to twenty years, the genetics study of apoptosis in the model organism *C. elegans, Drosophila melanogaster*, and the biochemistry study of apoptosis in mammals have provided an initial understanding of the molecular mechanisms of apoptotic cell clearance (Chen et al., 2010; Suzuki et al., 2013).

**FIGURE 2:**
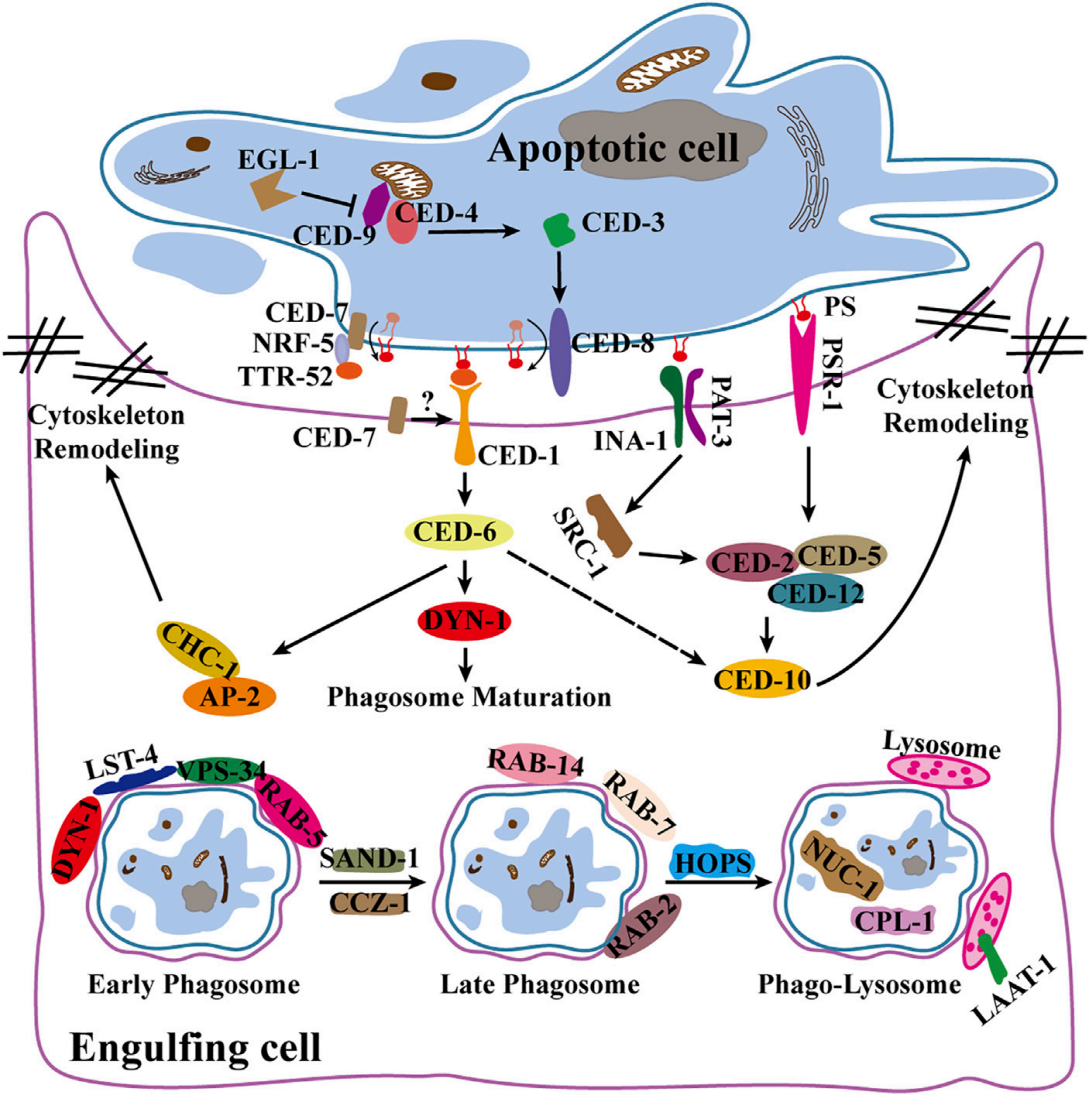
Apoptosis and apoptotic cell clearance pathways in *C. elegans*. Illustrates apoptogenesis, engulfment and phagosome maturation pathways and related signaling factors.

Apoptosis is determined by the activation of caspases and mitochondrial control pathways. Activation of pro-apoptotic factors (Bax, Bak, Bad, Bid, Puma, Bim, and Noxa) and anti-apoptotic factors (Bcl-2, Bcl-xL, Bcl-w, Mcl-1) within the Bcl-2 family determines whether apoptosis occurs in cells. When cells undergo apoptosis, the signal is transmitted to downstream caspases, and the inhibitor of apoptosis (IAP) family (XIAP, cIAP1, C-IAP2, NAIP, Livin and Survivin) prevents apoptosis by blocking the activity of various caspases (Silke and Meier, 2013; Rongvaux et al., 2014). The TNFR family (TNFR1/2, Fas and DR3/4/5) and related ligands (TNF-α, FasL, TRAIL, TWEAK) are death receptors that, which upon activation, promote the formation of the death-inducing signaling complex (DISC) and ultimately activate caspase-8 and caspase-3 (Chung et al., 2016). In the development of *C. elegans*, the key proteins involved in apoptosis are EGL-1, CED-9, CED-4, CED-3 and CED-8. CED-8 is cleaved by CED-3 caspase as its substrate, enhancing apoptotic activity and promoting PS ectopia in apoptotic cells (Chen et al., 2013a; Suzuki et al., 2013) (Figure 2).

Genetic screening in *C. elegans* identified two partially redundant phagocytic pathways (*ced-1/6/7* and *ced-2/5/10/12*) that are highly evolutionarily conserved (Reddien et al., 2001). CED-1 (Draper in flies, MEGF10 in mammals) (Zhou et al., 2001) interacts with CED-6 (dCed-6 in flies, GULP in mammals) (Liu and Hengartner, 1998; Su et al., 2002) to recruit the clathrin protein CHC-1 and its junctional components AP2 and epsin (Chen et al., 2013b; Shen et al., 2013), rearranging the cytoskeleton and facilitating phagocytic pseudopod expansion. CED-2, CED-5, CED-10, CED-12, and PSR-1 (homologs of CrkⅡ, DOCK180, RacGTPase, PH-SH3 domain, and PSR, respectively, in mammals) constitute another phagocytic pathway (Gumienny et al., 2001; Hsu and Wu, 2010; Wu et al., 2017). Phagocytes extend pseudopods for the enveloping of apoptotic cells to form phagosomes under the influence of two pathways. After maturation by a series of Rab GTPases, phagosomes fuse with lysosomes to form phagolysosomes (Guo et al., 2010). Finally, apoptotic cell fragments are degraded by lysosomal membrane proteins LAAT-1, as well as a variety of lysosomal hydrolases, including cathepsin L1(CPL-1) and type II DNA lyase (NUC-1) (Lyon et al., 2000; Liu et al., 2012; Xu et al., 2014) (Figure 2).

Apoptotic cell clearance plays an essential function in organ development and dynamic tissue homeostasis. Impaired apoptotic cell clearance has been linked to the development of various severe chronic inflammatory or catastrophic autoimmune disorders (e.g., systemic lupus erythematosus) as well as neurodegenerative diseases (e.g., Alzheimer’s disease).

### Ubiquitylation Regulates p53 in Apoptosis

**FIGURE 3:**
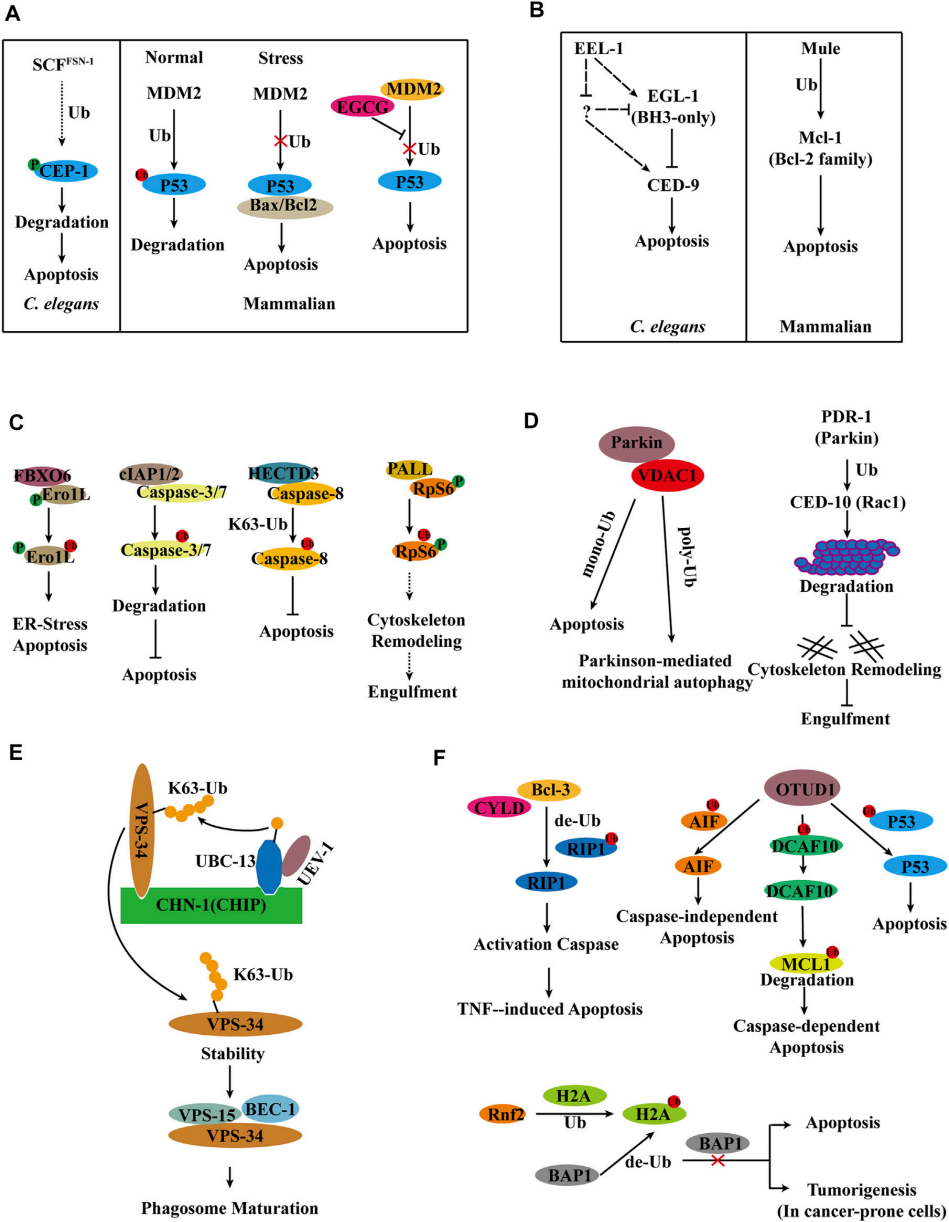
Model for E3s regulation of apoptosis and apoptotic cell clearance. **(A)** Ubiquitylation regulates p53 in apoptosis in *C. elegans* and Mammals. **(B)** E3 ligase Mule/EEL-1 as a regulator of apoptosis in *C. elegans* and Mammals. **(C)** Ubiquitylation regulates apoptosis in Mammals and *Drosophila*. **(D)** E3 ligase Parkin/PDR-1 in apoptosis and apoptotic cell clearance in *C. elegans* and Mammals. **(E)** UBC-13 and CHN-1 stabilize VPS-34 by ubiquitinating VPS-34 and promote phagosome maturation. **(F)** DUBs in apoptosis.

The p53 protein is mainly distributed in the nuclear plasma, binds specifically to DNA, identifies DNA damage sites, and monitors genomic integrity by post-translational changes such as phosphorylation, acetylation, methylation, and ubiquitination (Derry et al., 2001). Knockout of p53 in *mdm2* (E3 ubiquitin ligase) mutant mice avoid the embryonic lethal phenotype, and the level of p53 is significantly increased in *mdm2* mutant mice, therefore suggesting that MDM2 mediates the ubiquitinated degradation of p53 for stabilization of p53 levels. p53 is maintained at a low level by MDM2 in normal cells. When cells undergo stress, ubiquitination of p53 is suppressed and interacts with the anti-apoptotic proteins Bax and Bcl2 to induce apoptosis (Haupt et al., 1997; Brooks and Gu, 2006; Jain and Barton, 2010). Interestingly, epigallocatechin-3-gallate (EGCG) disrupts the interaction of p53 with MDM2 by interacting with the N-terminal structural domain (NTD) of p53, inhibits the ubiquitination of p53, induces apoptosis, and stabilizes the antitumor activity of p53 (Zhao et al., 2021) (Figure 3A). *C. elegans* contains a single member of p53 family, *cep-1*, which is regulated by Akt/PKB, Brca1, and the p53-binding protein iASPP and induces apoptosis in response to DNA damage in germ cells (Bergamaschi et al., 2003; Boulton et al., 2004; Quevedo et al., 2007). *C. elegans* RNAi screening revealed that Skp1/cullin/F-box (SCF) E3 ubiquitin ligase negatively regulates *cep-1*-dependent germ cell apoptosis in response to genotoxic stress. Among the six cullin genes in *C. elegans*, only *cul-1* regulates ENU-induced germ cell death. The Skp1-related gene *skr-1*, and the ring box genes *rbx-1* and *rpm-1*, negatively regulate cep-1-dependent germ cell apoptosis in response to the DNA-alkylating agent N-ethyl-N-nitrosourea (ENU). RNAi screening revealed that the FSN, F-box protein inhibits ENU-induced germ cell death. Deletion of *cep-1* overcompensates for the enhanced apoptotic phenotype observed in *fsn-1* mutants. In response to ENU, where endogenous CEP-1 is phosphorylated and regulated by FSN-1, IP experiments show that FSN-1 does not interact with CEP-1, and therefore FSN-1 may indirectly regulate CEP-1. SCF^FSN−1^ is also capable of directly binding and degrading phosphorylated CEP-1. Therefore, the E3 ubiquitin ligase SCF^FSN−1^ is a crucial regulator of CEP-1-dependent apoptosis (Gao et al., 2008) (Figure 3A).

### E3 Ligase Mule/EEL-1 as a Regulator of Apoptosis

The mitochondrial response to apoptotic stimuli induced by damaging signals (such as DNA damage) in mammalian cells is regulated by pro- and anti-apoptotic Bcl-2 family (Gross et al., 1999). Mcl-1, an anti-apoptotic member of the Bcl-2 family, is degraded at the onset of apoptosis, which is blocked by proteasome inhibitors in HeLa cells. IP ubiquitination experiments in HeLa cells have shown that Mcl-1 can undergo ubiquitination, and biochemical fractionation of cell extracts has identified Mule as the E3 ubiquitin ligase. When Mule expression is knockdown, Mcl-1 is stabilized, and DNA-damaging agent-induced apoptosis is reduced (Zhong et al., 2005) (Figure 3B). *C. elegans* sense DNA damage through the 9-1-1 complex (*hpr-9*, *hus-1*, and *mrt-2*) and *clk-2*, which are required to inhibit mitotic growth in germ cells and initiate *cep-1*-dependent apoptosis (Ahmed et al., 2001). A screen of 108 E3 ligases in *C. elegans* revealed that the HECT-domain E3 ligase EEL-1 (a homolog of human Huwe1/ARF-BP1/Mule) is involved in ionizing radiation (IR)-induced germ cell apoptosis. The number of apoptotic cells generated by IR-induced was significantly reduced in the *eel-1* mutant compared to the wild type. However, the *eel-1; ced-1* double mutants do not reduce the number of apoptotic cells in the *ced-1* mutants either in the germline or in the embryo; therefore, *eel-1* regulates DNA damage-induced germ cell apoptosis. Loss of *eel-1* has not suppressed the phenotype of a significant increase in the level of germ cell apoptosis in the *ced-9(n2812)* mutants, regardless of the presence or absence of damage. The combined data suggest that *eel-1* acts upstream or independently of *ced-9*. The exclusion of a change in CED-9 protein levels in *eel-1(ok1575)* or *eel-1(zu462)* mutants after IR treatment indicates that EEL-1 does not target CED-9 for degradation (Ross et al., 2011) (Figure 3B).

### FBXO6 Mediates the Ubiquitinated Degradation of Ero1L to Inhibit Endoplasmic Reticulum Stress-Induced Apoptosis

Endoplasmic reticulum stress is caused by an accumulation of unfolded or misfolded proteins in the endoplasmic reticulum, and endoplasmic reticulum stress can lead to cell death (Sano and Reed, 2013; Chen et al., 2014). An unbiased screen reveals that FBXO6 interacts with glycosylated Ero1L. FBXO6 encodes a member of the F-box protein family involved in phosphorylation-dependent ubiquitination. Ero1L, a transcriptional target of CHOP that plays a crucial role in endoplasmic reticulum stress-induced apoptosis, was found to be significantly elevated in A549 cells where FBXO6 expression was silenced by shRNA. The dual knockdown of FBXO6 and Ero1L rendered A549 cells less susceptible to endoplasmic reticulum stress-induced apoptosis. Thus, FBXO6 regulates endoplasmic reticulum stress-induced apoptosis by targeting the degradation of Ero1L ubiquitination (Chen et al., 2016) (Figure 3C).

### Ubiquitination of Caspase-8 by the E3 Ubiquitin Ligase HECTD3 Through the K63-Linked Ubiquitin Chain Inhibits Caspase-8 Activation

Caspases, a family of cysteine proteases, are prevalent death effector molecules. The activation of caspases-8 by active caspase-3/7 induces apoptosis. cIAP1/2, an apoptosis inhibitor, binds to caspases-3/7 and promotes their ubiquitination for proteasome-dependent degradation, thereby partially inhibiting apoptosis (Huang et al., 2000; Choi et al., 2009). It has been discovered that the E3 ligases SIAH2 and POSH inhibit caspase-8 function, but it is unknown if these E3 ligases ubiquitylate caspase-8. Subsequently, it has been demonstrated that the E3 ubiquitin ligase HECTD3 interacts with the DED domain of caspase-8 *via* the DOC domain but not with caspase-3/7. Experiments in 293T cells reveal that overexpression of HECTD3 increases ubiquitination of caspase-8 and that HECTD3 ubiquitinates caspase-8 by K63-linked ubiquitination. Apoptosis and caspase-8 activation induced by an exogenous pathway is enhanced in the absence of HECTD3, whereas HECTD3 inhibits caspase-8 activation. HECTD3 promotes cell survival *via* ubiquitination of caspase-8 and breast cancer-associated overexpression (Li et al., 2013) (Figure 3C).

### E3 Ligase PALL-SCF Complex Mediates Ubiquitin Degradation of Phosphorylated RpS6 to Promote Apoptotic Cell Clearance

A genome-wide RNAi screen of *Drosophila* Schneider S2 cells reveals that PALL, an F-box protein that functions in the SkpA/DCullin-1/F-Box (SCF) complex, affects phagocytosis (Silva et al., 2007). IP mass spectrometry of overexpressed PALL in S2 cells identified the protein RpS6 with which it interacts, and the necessity of RpS6 phosphorylation for maintaining their interactions. PALL promotes ubiquitinated degradation of RpS6. In *Drosophila*, the RpS6 loss-of-function mutation suppresses the loss-of-function phenotype of *pall*, but overexpression of RpS6 results in a phagocytosis deficiency, indicating that RpS6 plays a negative regulatory role in phagocytosis. PALL ubiquitination of RpS6 leads to upregulated RAC2 GTPase activation and F-actin remodeling, enhances phagocytosis, and determines its specific involvement in efferocytosis is (Xiao et al., 2015) (Figure 3C).

### SLI-1, an E3 Ligase Inhibits Engulfment of Apoptotic Cells in *C. elegans*

Deletion of *sli-1* in *C. elegans* suppressed the phagocytosis-deficient phenotype generated by mutations in the phagocytosis *ced* gene, but did not affect the increased apoptosis phenotype caused by mutations in the apoptogenesis *ced* gene. CBL, a worm SLI-1 homolog, interacts with Rac, Crk, and ABL kinases in mammals (Soubeyran et al., 2003). Genetic analysis reveals that SLI-1 functions in engulfment, however, it is independent of the two well-characterized engulfment pathways, as well as the ABL-1 pathway. Overexpression of various truncated forms of SLI-1 in *sli-1* mutants uncovers that SLI-1 regulates phagocytosis through its TKB domain. Deficiency of *sli-1* inhibits the DTC migration defect of the *ced-10* Rac pathway but not the phagocytosis defect of the *ced-10* Rac pathway. Thus, *sli-1* suppresses apoptotic cell engulfment by a mechanism independent of its E3 ligase function, showing that other cell biological processes are involved in the engulfment of apoptotic cells (Anderson et al., 2012).

### E3 Ligase Parkin/PDR-1 in Apoptosis and Apoptotic Cell Clearance

Interactions between Parkin and Rac1 have been shown to be engaged in cytoskeletal rearrangements in aging human brains. CED-10 (a mammalian ortholog of Rac1) is involved in cytoskeletal rearrangement and apoptotic cell clearance in *C. elegans* (Sun et al., 2012). In mammals, rac1 is degraded by ubiquitination modification when it is active. Parkin, a mammalian E3 ligase, is involved in the stability of cytoskeletal components. In *ced-10* mutants, the absence of the E3 ligase PDR-1 (a homolog of mammalian Parkin) decreases the number of apoptotic cells. Additionally, when the proteasome’s function is inhibited with MG-132, the number of apoptotic cells is decreased in *ced-10* mutants. Overexpression of CED-10, PDR-1, and Ub (wt or K48R) in 293T cells to detect the ubiquitination level of CED-10 following IP revealed that PDR-1 is required for CED-10 ubiquitination. And the deleting of *pdr-1* increases the amount of CED-10 protein in *C. elegans*. PDR-1 inhibits phagocytosis through modulating the ubiquitination of CED-10 (Cabello et al., 2014) (Figure 3D).

Unlike the mechanism of action of PDR-1 in apoptotic cell clearance in *C. elegans*, Parkin-mediated ubiquitination of VDAC1 in mammals promotes both mitochondrial autophagy and apoptosis (Matsuda et al., 2010). Overexpression of VDAC1 (WT, mono-KR, poly-KR, and all-KR) in VDAC1 KO mouse embryonic fibroblasts reveals that Parkin ubiquitinates VDAC1 in two distinct ways, poly-ubiquitination and mono-ubiquitination, and that poly-ubiquitination deficient VDAC1 prevents apoptosis while mono-ubiquitination deficient VDAC1 induces apoptosis. Porin, a *Drosophila* homolog of mammalian VDAC1, has ubiquitin-deficient mutations that cause locomotor abnormalities, apoptosis, and neurodegeneration. The T415N mutation in Parkin leads to a monoubiquitination defect in VDAC1, and overexpression of Parkin T433N (T415N of Parkin in PD patients) in the *Drosophila park* ^*1*^ mutant does not rescue the phenotype of the mutant (Ham et al., 2020) (Figure 3D).

### UBC-13-UEV-1-CHN-1 Ubiquitinates of VPS-34, Thereby Stabilizing it and Promoting Phagosome Maturation

VPS-34 (a class III PI3-kinase) and PIKI-1 (a class II PI3-kinase) function synergistically in the production of PtdIns3P on phagosomes, where PIKI-1, in conjunction with MTM-1, regulates the level of PtdIns3P to maintain pseudopod extension and phagosome closure. VPS-34 produces PtdIns3P during the sealing stage and ultimately regulates phagosome maturation. *ubc-13* was identified as the mutant associated with apoptotic cell clearance by genetic screening. Ubc13, an E2 ubiquitin-conjugating enzyme, cooperates with the noncatalytic E2-like partner protein Mms2 or Uev1A to mediate k63-linked polyubiquitination of multiple substrates. In *C. elegans*, UBC-13 plays a critical role by accelerating the K63-mediated clearance of maternal membrane proteins (Sato et al., 2014). Furthermore, the deletion of *ubc-13* has been shown to alter the maturation of phagosomes using 2XFYVE and RAB-5 label rate statistics.

Yeast two-hybrid screening revealed that the E3 ubiquitin ligase CHN-1, homologous to human CHIP, interacts with UBC-13.VPS-34 interacts with the TPR domain of CHN-1 through the C2 domain. Genetic analysis revealed that VPS-34 affects phagosome maturation through the same signaling pathway as UBC-13, UEV-1, and CHN-1. *In vitro* and *in vivo* Ubiquitination experiments demonstrates that UBC-13, UEV-1 (E2 variant), and CHN-1 mediate the K63-linked poly-ubiquitination of VPS-34. Further experiments indicate that K63-linked poly-ubiquitination of VPS-34 promotes VPS-34 stability, which is inconsistent with the degradation function of poly-ubiquitination. Additional investigations reveal that modifying VPS-34 with ubiquitin can enhance the stability of VPS-15 and influence the formation of the VPS-34 complex. Overall, UBC-13 and CHN-1 ubiquitinate VPS-34 and cooperate in PtdIns3P-mediated cellular processes regulated by VPS-34 to promote apoptotic cell clearance in *C. elegans* (Liu et al., 2018) (Figure 3E).

### Deubiquitinating Enzymes in Apoptosis

Excessive apoptosis of hepatocytes causes various liver diseases, and defective tumor necrosis factor (TNF)-induced apoptosis correlates with autoimmune diseases and liver diseases (Nagaki et al., 2000). RIP1’s ubiquitinated status plays a crucial role in the formation of complex II, which regulates TNF-induced caspase activation. Bcl-3 has been discovered to facilitate the development of the death-inducing complex II by promoting RIP1 deubiquitination in a CYLD-dependent manner. This complex further activates the caspase cascade, hence inducing apoptosis (Hu et al., 2022) (Figure 3F).

In normal cells, AIF located in mitochondria and participated in the maintenance of oxidative phosphorylation. However, when cells are stimulated externally, AIF moves from mitochondria to the nucleus, binds non-specifically to DNA, and induces caspase-independent apoptosis. The study reveals that OTUD1 binds directly to AIF and mediates the deubiquitination of AIF lysine sites 244 and 255, thereby triggering caspase-independent apoptosis. Additionally, OTUD1 improves the stability of DCAF10 by deubiquitination. DCAF10, a component of the CUL4-DDB1 ubiquitin ligase complex, mediates the ubiquitinated degradation of MCL1. Thus, OTUD1 indirectly promotes the degradation of MCL1 and initiates caspase-dependent apoptosis (Luo et al., 2021). In addition to these functions, OTUD1 interacts directly with p53 and deubiquitinates p53. The deubiquitination activity of OTUD1 is required for the stability of p53 *via* an inactive OTUD1 mutant (C320S OTUD1 mutant) (Piao et al., 2017) (Figure 3F). It has been confirmed that overexpression of OTUD1 increases the cleavage of caspase-3 and PARP, leading to an increase in apoptosis.

Humans and animals utilize the deubiquitinating enzyme BAP1 as a tumor suppressor (Wiesner et al., 2011). A genome-wide CRISPR-Cas9 screen reveals that apoptosis mediated by Bap1 deletion or inactivation is predominantly regulated by Rnf2, an E3 ubiquitin-linked enzyme responsible for attaching a ubiquitin at lysine 119 of histone H2A. Moreover, Bap1 specifically eliminates the ubiquitin added by Rnf2. Deletion of Bap1 increases H2AK119 ubiquitination and decreases cell viability. In conclusion, these results demonstrate that whereas many cells do not survive in the absence of Bap1 due to activation of the apoptotic pathway, Bap1 deletion does not cause apoptosis in some cancer-prone cells, such as melanocytes, but rather promotes carcinogenesis (He et al., 2019) (Figure 3F).

## Conclusion

Ubiquitination is associated with a wide variety of cellular functions and practically all aspects of growth and development. Numerous signaling pathways and genes are involved in ubiquitination, with E3 being one of the most extensively investigated enzymes. Apoptosis, which is highly conserved evolutionary, is essential for the organism’s development and homeostasis, enabling it to avoid inflammatory and immune-related disorders. E3 ligases have been discovered to be engaged in the complicated series of apoptogenesis and clearance in mammals; and regulates several key molecules involved in the apoptotic pathway. Although the role of E3 ligases in apoptotic regulation was initially recognized in mammals, the specific mechanism by which E3 ligases influence apoptotic regulation remains a mystery due to a large number of E3 species.

This article discusses the ubiquitination pathway and apoptotic cell clearance, as well as the significance of ubiquitination in apoptosis and apoptotic cell clearance. Additionally, *C. elegans* and mammals, are highly conserved in terms of ubiquitination modifications and apoptotic cell clearance. Due to the model organism’s outstanding genetic and cell biology as well as other research tools, the research in lower creatures (*C. elegans* and *Drosophila*) has paved the way for the study of mammals and established a biological basis for ubiquitination- and apoptosis-mediated inflammatory or immunological disorders.

Research on the ubiquitination mechanisms during apoptosis and apoptotic cell clearance is still quite limited. Remarkably, little is known about the regulation of ubiquitination during the clearance of apoptotic cells. Future studies will use model organisms, such as *C. elegans* and *Drosophila*, to elucidate the molecular mechanisms underlying this process. Ubiquitination is a dynamic and reversible modification, and substrate deubiquitination requires deubiquitinating enzymes. However, little is known about the function of deubiquitinating enzymes in apoptosis and the clearance of apoptotic cells. Since DUBs play a critical role in cell differentiation, neurological diseases, and transcriptional regulation, future studies utilizing *C. elegans* and *Drosophila* to investigate the role of DUBs in apoptosis essential factors will also contribute to our understanding of apoptosis mechanisms. We will uncover other complex interactions and regulatory mechanisms between ubiquitination processes (especially E3s and DUBs) and apoptotic clearance in *C. elegans*, *Drosophila*, and mammals, thereby providing additional molecular mechanisms for inflammation, neurological disorders, and autoimmune diseases.
